# Circulating Monocytic Myeloid-Derived Suppressor Cells Are Elevated and Associated with Poor Prognosis in Acute Myeloid Leukemia

**DOI:** 10.1155/2020/7363084

**Published:** 2020-12-21

**Authors:** Huiping Wang, Qianshan Tao, Zhitao Wang, Qing Zhang, Hao Xiao, Mei Zhou, Yi Dong, Zhimin Zhai

**Affiliations:** Department of Hematology, The Second Hospital of Anhui Medical University, and Hematology Research Center, Anhui Medical University, Hefei, Anhui, China

## Abstract

**Background:**

Monocytic myeloid-derived suppressor cells (M-MDSCs) characterized with the phenotype of CD14^+^HLA-DR^low/-^ have attracted a lot of attention in the field of human tumor immunology. However, little is known about the roles of M-MDSCs in acute myeloid leukemia (AML) as opposed to their multiple roles in solid tumors.

**Methods:**

We examined the frequencies of M-MDSCs identified for CD14^+^HLA-DR^low/-^ by flow cytometry in the peripheral circulating blood of 109 newly diagnosed adult patients with AML and 30 healthy controls (HC). Then, we, respectively, validated the clinic significance of circulating M-MDSCs on the relevance of spectral features for diagnostic stratification, induction therapy response, treatment effect maintenance, and long-term survival in AML.

**Results:**

Circulating M-MDSC frequencies of AML were significantly higher than those of HC both in CD14^+^ monocytes (46.22% ± 2.95% vs. 1.07% ± 0.17%, *p* < 0.01) and peripheral blood mononuclear cells (PBMCs) (4.21% ± 0.80% vs. 0.17% ± 0.03%, *p* < 0.01). Elevated circulating M-MDSCs in patients with AML were significantly associated with low complete remission (CR) rate, high relapse/refractory rate, and poor long-term survival, but had no correlation with common clinic risks and cytogenetic molecular risk categories.

**Conclusions:**

It was demonstrated that circulating M-MDSCs are elevated and associated with poor prognosis in AML, suggesting M-MDSCs might be a prognostic indicator for AML.

## 1. Introduction

Acute myeloid leukemia (AML) is a hematopoietic malignancy characterized by an accumulation of undifferentiated and functionally heterogeneous populations of leukemic blasts. Recently, a large number of evidences suggest that the proliferation, survival, and drug resistance of leukemic blasts are modulated by the tumor microenvironment (TME), which are composed of a variety of cells, extracellular matrix, and extracellular molecules. Especially, several individual immune cell subsets and immune molecules are also involved in the TME of AML [1]. CD14-positive monocytes with low HLA-DR or no HLA-DR expression (CD14^+^HLA-DR^low/-^ monocytes) have been grouped into a larger class of immunosuppressive cells called monocytic myeloid-derived suppressor cells (M-MDSCs) [2–5]. M-MDSCs have attracted a lot of attention in the field of human tumor immunology, and their functional capacities related to the immunosuppressive mechanisms have been illustrated in recent years [4, 6]. However, little is known about the roles of M-MDSCs in hematopoietic malignancy especially for AML as opposed to their multiple roles in solid tumors [7].

So far, only some studies have reported that the frequencies of M-MDSCs are higher in the peripheral blood of AML than that of healthy controls (HC) [8, 9]. Those M-MDSCs may be derived from leukemic or apparently normal progenitors [8] and inhibit the activity of CD8 T cell [9]. But the clinical significance and related influencing factors of M-MDSCs in AML is not clear. In the present study, we not only systematically detected the frequencies of M-MDSCs in the peripheral circulating blood of 109 newly diagnosed adult patients with AML but also evaluated the frequencies of circulating M-MDSCs and their correlation with various clinical parameters, short-term efficacy, and long-term survival in the pathogenesis of leukemia. Finally, we found that circulating M-MDSCs are significantly elevated and might predict undesirable clinic outcomes and poor long-term prognosis in AML.

## 2. Methods

### 2.1. Patients and Samples

This study included 109 newly diagnosed adult patients with AML and age-/sex-matched 30 healthy adult controls in the Second Hospital of Anhui Medical University between 2013 and 2019. All patients were characterized by their own diagnostic stratification and received standard induction chemotherapy followed by either consolidation therapy or allogeneic hematopoietic stem cell transplantation (allo-HSCT) in accordance with the Chinese guidelines for diagnosis and treatment of adult AML [10, 11]. The detailed clinical characteristics of a total of 109 patients with AML are shown in Table 1. The samples of peripheral blood from all patients and controls were collected prior to any treatment and were evaluated within 6 hours. This study was approved by the Institutional Review Board Institutional of the Second Hospital of Anhui Medical University.

### 2.2. M-MDSCs Analysis

Peripheral blood mononuclear cells (PBMCs) were stratified on FicollHypaque (Amersham Biosciences, Sweden) and separated by centrifugation for 25 min (500 g). Subsequently, PBMCs were collected and washed with phosphate-buffered saline (PBS). After washing, 100 *μ*L PBMCs was incubated with monoclonal antibodies and analyzed by flow cytometry Cytomics® FC500 (Beckman Coulter, Miami, FL, USA), and EXPO 32 Multicomp software was used for data acquisition and analysis. The following monoclonal antibodies were purchased from Beckman Coulter (Miami, FL, USA): FITC-labeled CD14 (clone RMO52), PE-labeled HLA-DR (clone Immu-357), and PC5-labeled CD45 (clone J.33). M-MDSCs subsets were stained and identified by the phenotype of CD14^+^HLA-DR^low/-^ in CD45^++^ cells. Isotype-matched controls were included in the experiments and were used to define the cutoff point for positive/negative staining.

### 2.3. Statistical Analysis

All statistical analysis was performed with SPSS 16.0 (IBM, Chicago, IL, USA). The Student's *t* test, chi-squared test, and Fisher's exact probability tests were used as appropriate to evaluate the significance of the differences in data between different groups. If variances within groups were not homogeneous, a nonparametric Mann–Whitney test was used. Overall survival (OS) was used and defined as the time from date of diagnosis until the date of death. The prognostic value was evaluated by Kaplan-Meier survival curves. We used healthy adult controls as an indicator to generate the ROC curve and determined the optimal cutoff value based on the Yoden index. The cutoff values of M-MDSCs were 0.5%. More than or equal to the respective cutoff value was defined as the high-frequency group, and less than the respective cutoff value was defined as the low-frequency group. The *p* value less than 0.05 was considered statistically significant.

## 3. Results

### 3.1. Circulating M-MDSCs Were Elevated in AML

**Figure 1:**
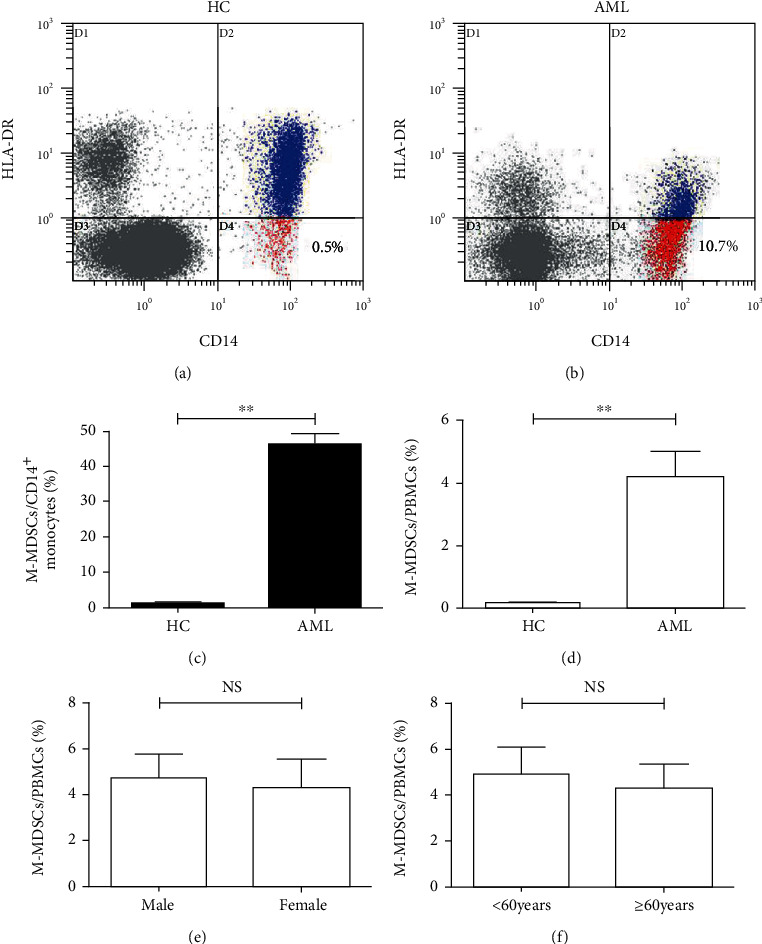
The frequencies of circulating M-MDSCs were elevated in AML. Representative flow cytometry dot plots demonstrated the frequencies of CD14^+^HLA-DR^LOW/-^ M-MDSCs in AML (a) and HC (b). The frequencies of circulating M-MDSCs of AML (*n* = 109) and HC (*n* = 30) in the percentage of CD14^+^ monocytes (c) and PBMCs (d). The frequencies of circulating M-MDSCs of AML in different gender groups (e) and age groups (f). AML: acute myeloid leukemia; HC: healthy controls; NS: not significant; ^∗∗^*p* < 0.01.

The frequencies of M-MDSCs in 109 newly diagnosed adult patients with AML and 30 HC were investigated by flow cytometry in the present study. We found that circulating M-MDSCs frequencies of AML were significantly higher than those of HC both in CD14^+^ monocytes (46.22% ± 2.95% vs. 1.07% ± 0.17%, *p* < 0.01; Figures 1(a) and 1(c)) and PBMCs (4.21% ± 0.80% vs. 0.17% ± 0.03%, *p* < 0.01; Figures 1(b) and 1(d)). Thus, we alternatively used the frequencies of M-MDSCs in PBMCs for further analysis in the following text. However, no significant differences in the circulating M-MDSCs frequencies were found in the different gender and age groups (Figures 1(e) and 1(f)). Together, it was suggested that circulating M-MDSCs were elevated in AML, which was not affected by age and gender.

### 3.2. Circulating M-MDSCs Were Associated with Poor Prognosis in AML

**Figure 2:**
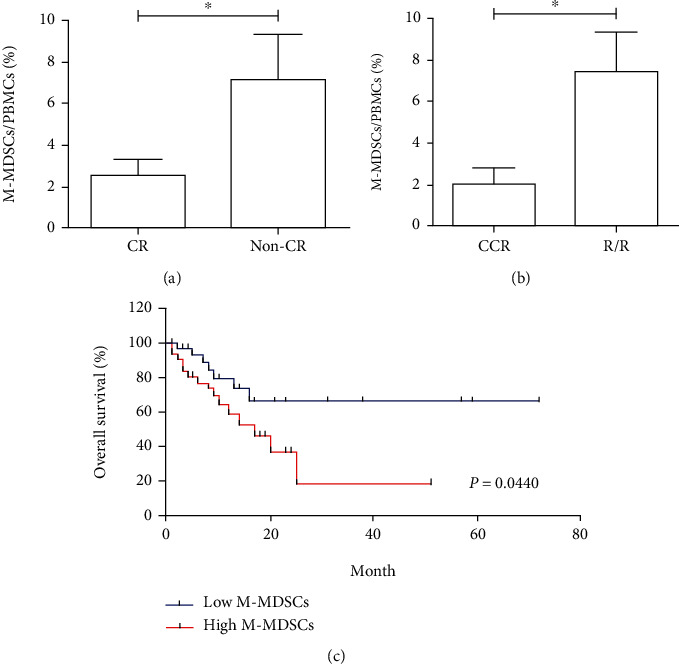
The prognostic relevance of circulating M-MDSCs in AML. The frequencies of circulating M-MDSCs in the CR group were significantly lower than the non-CR group (a). The frequencies of circulating M-MDSCs in the CCR group were significantly lower than the R/R group (b). The low circulating M-MDSC frequency group had a significant survival advantage compared with the high group (c).^∗^*p* < 0.05.

62 cases (57%) of 109 patients with AML received standard treatment consisting of one or two cycles of induction chemotherapy followed by either consolidation therapy or allo-HSCT; the other 47 cases (43%) only received supportive care because of their old age or poor physical condition. In Table 1 and Figure 2(a), 35 of 62 cases (56%) obtained complete remission (CR) and 27 of 62 cases (44%) did not obtain CR (non-CR) after the first induction chemotherapy. The frequencies of circulating M-MDSCs in the CR group (2.54% ± 0.75%) were significantly lower than the non-CR group (7.08% ± 2.29%, *p* < 0.05). In Table 1 and Figure 2(b), 27 of 62 cases (44%) preserved continued complete remission (CCR) and 35 of 62 cases (56%) eventually became refractory or relapsed (R/R) disease after the reinduction therapy or consolidation therapy/allo-HSCT. The frequencies of circulating M-MDSCs in the CCR group (2.03% ± 0.76%) were significantly lower than the R/R group (7.47% ± 1.77%, *p* < 0.05). For follow-up analyses, a total of 62 cases that received standard treatment were included. As shown in Figure 2(c), patients with the low circulating M-MDSCs frequency had a significant survival advantage than patients with the high circulating M-MDSCs frequency (*p* = 0.0440). Together, it was suggested that elevated circulating M-MDSCs at the time of new diagnosis were a prognostic indicator to predict poor outcomes in patients with AML.

### 3.3. Circulating M-MDSCs Were Not Correlated with Common Clinic Risks in AML

**Figure 3:**
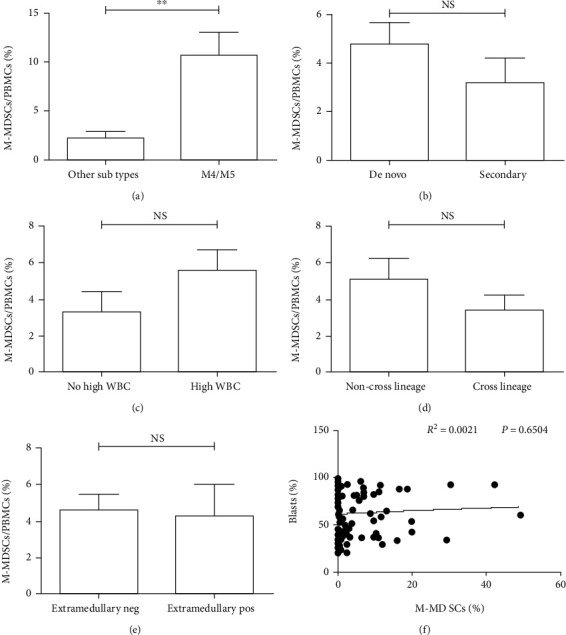
The correlation of circulating M-MDSC frequency and clinic risk categories in AML. The frequencies of circulating M-MDSCs in FAB M4/M5 subtype groups were significantly higher than the other FAB subtype groups (a). No significant statistical differences on circulating M-MDSCs were found in the different groups of AML type, WBC count, cross linage expression, extramedullary infiltration, and leukemic blasts percentage (b–f). NS: not significant; ^∗∗^*p* < 0.01.

In this article, we analyzed the correlation between the frequency of circulating M-MDSCs and common clinical risks in all 109 AML cases. In Figure 3(a), our data demonstrated that the frequencies of circulating M-MDSCs in French-American-British (FAB) classification system acute monocytic leukemia (M4)/acute myelomonocytic leukemia (M5) subtype groups were significantly higher than those in other FAB subtype groups (10.74% ± 2.29% vs. 2.29% ± 0.53%, *p* < 0.01). However, in Figures 3(b)–3(f), there were no significant statistical differences in circulating M-MDSCs in the other groups of AML type including de novo AML and secondary AML, white blood cell (WBC) count, cross linage expression, extramedullary infiltration, and leukemic blasts percentage (all *p* > 0.05). Altogether, these data showed no correlation between circulating M-MDSCs and common clinic risks except for the FAB subtype.

### 3.4. Circulating M-MDSCs Were Not Correlated with Cytogenetic Molecular Risk Categories in AML

**Figure 4:**
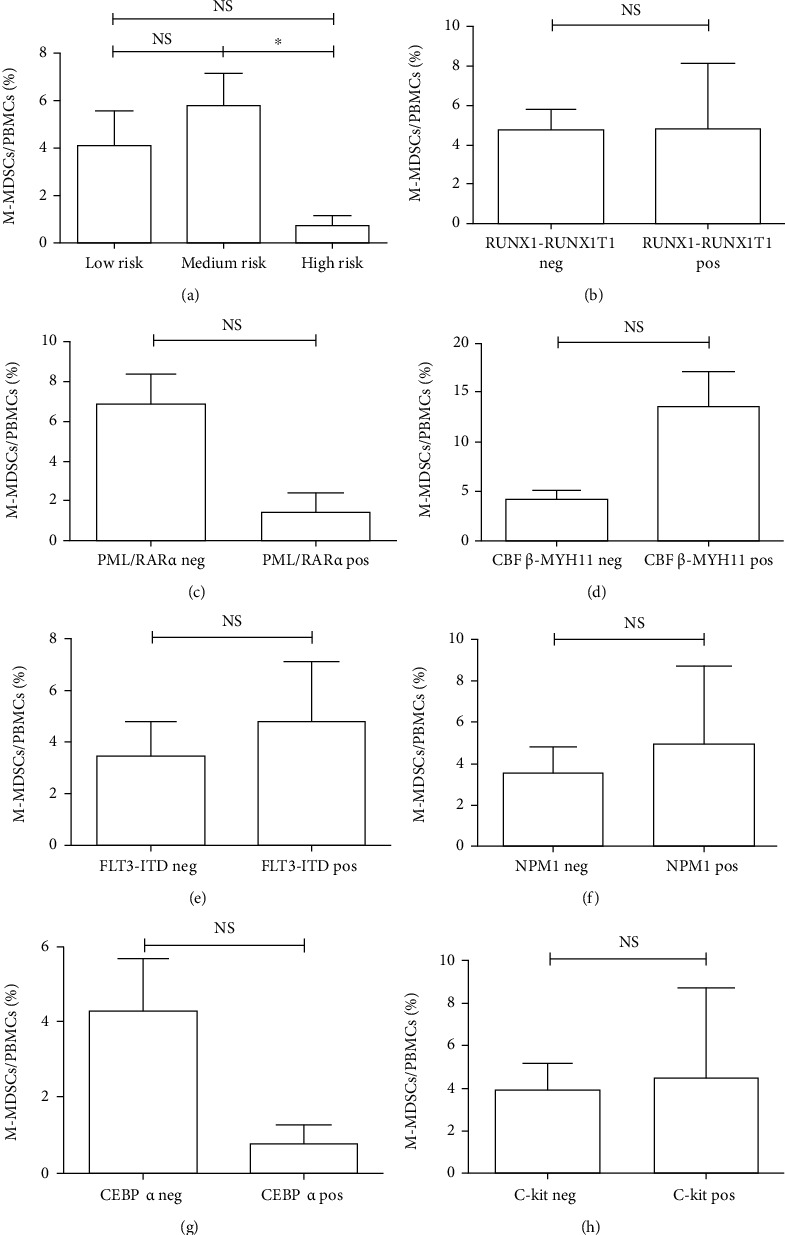
The correlation of circulating M-MDSC frequency and cytogenetic/molecular risk categories in AML. No significant statistical differences in circulating M-MDSCs were found in the different groups of karyotype stratification, fusion gene, and gene mutation (a–h). NS: not significant.

In the present study, 109 newly diagnosed patients with AML were enrolled. In Table 1, 90 cases (83%) were detected of G-banded chromosome recognition and divided into the different groups of low risk, medium risk, and high risk, according to the Chinese guidelines for diagnosis and treatment of adult AML. 92 cases (84%) were detected of 44 kinds of fusion genes. 40 cases (37%) were detected of FLT3-ITD, NPM1, CEBPA, and C-kit gene mutations. Then, we analyzed the correlation between the frequency of circulating M-MDSCs and karyotype stratification, fusion gene, and gene mutation. We found there were no significant statistical differences on circulating M-MDSCs in the groups of karyotype stratification, fusion gene, and gene mutation (Figures 4(a)–4(h), all *p* > 0.05). Altogether, these data showed no correlation between circulating M-MDSCs and common cytogenetic molecular risk categories.

## 4. Discussion

In the late 1990s, Gr1^+^CD11b^+^ cells were suggested as defining the immunesuppressive myeloid cells which were functionally distinct from monocytes and neutrophils in mice [12, 13]. The unique name “myeloid-derived suppressor cells (MDSCs)” of these immunesuppressive myeloid cells was proposed in 2007 [2]. In humans, monocytes that have diminished or not HLA-DR expression have emerged as important mediators of tumor-induced immunosuppression [14]. So, CD14^+^HLA-DR^low/-^ monocytes have been grouped into a larger class of MDSCs and are commonly referred to as a special subtype of monocytic myeloid-derived suppressor cells (M-MDSCs) [14–16].

In recent years, great progress has been made in understanding the roles of M-MDSCs in human malignant tumors. Studies show that M-MDSCs are closely related to the tumor progression and treatment effect of patients with solid tumors [17, 18]. For example, it has been reported that the frequencies of M-MDSCs are significantly increased in small-cell lung cancer, head and neck tumor, and bladder cancer, and closely related to the clinical stage of the tumor [19–21]. In patients with hematological malignancies, the researches on M-MDSCs are mostly focused on lymphoma and myeloma [7, 22]. In our previous studies, the result has shown that M-MDSCs are elevated and correlated with tumor progression, and even as an indicator for the efficacy of therapy in patients with myeloma. Meanwhile, we have reported that the plasma cells are able to induce the accumulation of M-MDSCs in vitro [23]. However, studies on M-MDSCs have been relatively limited in leukemia especially AML, not alike in solid tumors and lymphoma as well as myeloma.

AML is a heterogeneous hematopoietic malignancy that derives from aberrant proliferation and accumulation of leukemic blasts. Immunological disorders of several individual immune cell subsets and immune molecules have been shown to be related to the pathogenesis of AML [1]. In our previous studies, we have reported that tumor immune escape mechanism mediated by CD4^+^CD25^+^ regulatory T cells (Tregs) and inhibitory cytokine IL-35 is a key factor for the proliferation and apoptosis of leukemic blasts [24, 25].

In this study, we further determined the proportion of M-MDSCs by the analysis of flow cytometry in the peripheral circulating blood of 109 newly diagnosed adult patients with AML and 30 matched HC. Compared with the HC group, we found that circulating M-MDSCs frequencies both in CD14^+^ monocytes and PBMCs were significantly increased in the AML group. Additionally, no significant difference in the proportion of circulating M-MDSCs was found in the different age and gender groups. So, it was illustrated that circulating M-MDSCs were elevated in AML, which might complicate with a severe immune dysfunction and contribute to patients' overall immune suppression.

Regarding the clinical values of M-MDSCs, our study showed that circulating M-MDSCs frequencies were significantly higher in the poor outcome patients with AML after the first induction of chemotherapy. The patients with high circulating M-MDSCs frequencies had a significantly shorter survival within 72 months of follow-up. These data were consistent with the same results of the study in solid tumor and lymphoma as well as myeloma [18, 26, 27]. It was suggested that elevated circulating M-MDSCs might be regarded as a prognostic indicator to predict the poor outcome in patients with AML. Moreover, we also analyzed the correlation between the frequency of M-MDSCs and common clinical and laboratory risk factors in patients with AML. We found that the high circulating M-MDSCs were only corrected to FAB M4/M5 subtypes mightily due to the increased proportion of monocytes in those special subtype patients. However, no other statistical correlation was found in AML types, WBC count, cross linage expression, extramedullary infiltration, leukemic blasts percentage, karyotype stratification, fusion gene, and gene mutation. Therefore, it was implied that M-MDSCs might be an independent prognostic indicator in AML.

In summary, circulating M-MDSCs were significantly elevated and closely related to the poor outcome of induction therapy in patients with AML. Circulating M-MDSCs could be used as a prognostic indicator to evaluate the survival of patients with AML. Targeting therapies for M-MDSCs might help to improve the host's antitumor immune function, which might be expected to become a new strategy for AML treatment in the future.

## Figures and Tables

**Table 1:** Characteristics of 109 newly diagnosed patients with AML.

Group	Subgroup	Number	Group	Subgroup	Number
Gender	Male	62	Cross lineage	Yes	37
	Female	47		No	72
Age	<60 y (14-59)	58	Karyotype	Low risk	28
	≥60 y (60-91)	51		Medium risk	47
FAB classification	M0	1		High risk	15
	M1	4		No data	19
	M2	33	Fusion gene	RUNX1-RUNX1T1	9
	M3	19		PML/RAR*α*	16
	M4	20		CBF*β*-MYH11	5
	M5	9		Others	79
	M6	1	Gene mutation	FLT3-ITD	9
	Mu	22		CEBPA	6
WBC	Low	24		NMP1	5
	Middle	26		C-kit	3
	High	59		Others	86
AML type	De novo	93	First induction	CR	35
	Secondary	16		Non-CR	27
Extramedullar	Yes	13	Curative effect	CCR	27
	No	96		R/R	35

Note: AML: acute myeloid leukemia; FAB: Franch-American-Britain; Mu: unclassifiable AML in morphology; WBC: white blood cell; CR: complete remission; Non-CR: not complete remission; CCR: continued complete remission; R/R: relapse/refractory.

## Data Availability

The data used to support the findings of this study are available from the corresponding author upon request.
